# Challenges to acquire similar learning outcomes across four parallel thematic learning communities in a medical undergraduate curriculum

**DOI:** 10.1186/s12909-023-04341-x

**Published:** 2023-05-18

**Authors:** Yan Zhou, Thomas H. Wieringa, Jasperina Brouwer, Agnes D. Diemers, Nicolaas A. Bos

**Affiliations:** 1 Key Laboratory of Intelligent Education Technology and Application of Zhejiang Province, Zhejiang Normal University, Jinhua, China; 2 Center for Education Development and Research in Health Professions (CEDAR), LEARN, University Medical Center Groningen, University of Groningen, Groningen, The Netherlands; 3 Department of Epidemiology, University of Groningen, University Medical Center Groningen, Groningen, the Netherlands; 4 Medical Decision Making, Department of Biomedical Data Sciences, Leiden University Medical Center, Leiden, The Netherlands; 5 Educational Sciences, Faculty Behavioural and Social Sciences, University of Groningen, Groningen, The Netherlands; 6 Wenckebach Institute for Education and Training, University of Groningen, University Medical Center Groningen, Hanzeplein 1, P.O.Box 30.001, 9700 RB Groningen, The Netherlands

**Keywords:** Competency-based medical education, Thematic learning communities, Problem-based learning

## Abstract

**Background:**

To train physicians who are able to meet the evolving requirements from health care, the University of Groningen Medical Center adopted in 2014 a new curriculum named G2020. This curriculum combines thematic learning communities with competency-based medical education and Problem-based learning. In the learning community program, different learning tasks were used to train general competencies. The challenge of this program was whether students acquire similar levels of learning outcomes within the different variations of the program.

**Method:**

We used the assessment results of three cohorts for the first two bachelor years. We used progress tests and written tests to analyze knowledge development, and the assessment results of seven competencies to analyze competence development. Concerning knowledge, we used the cumulative deviation method to compare progress tests and used the Kruskal–Wallis *H* test to compare written test scores between programs. Descriptive statistics are used to present all assessments of the students’ competencies.

**Results:**

We observed similarly high passing rates both for competency and knowledge assessments in all programs. However, we did observe some differences. The two programs that focused more on competencies development underperformed the other two programs on knowledge assessment but outperformed on competencies assessment.

**Conclusion:**

This study indicates that it is possible to train students in different learning programs within one curriculum while having similar learning outcomes. There are however some differences in obtained levels between the different programs. The new curriculum still needs to improve by balancing variations in the programs and comparability of assessments across the programs.

**Supplementary Information:**

The online version contains supplementary material available at 10.1186/s12909-023-04341-x.

## Background

Health care is becoming more complex in the twenty-first century: globalization, imbalanced workforce, expanding knowledge, technology development, patient empowerment, and increasing multidisciplinary collaboration are some of the challenges [1–3]. This complexity requires that medical trainees become specialists in terms of specific knowledge but simultaneously they need to acquire general professional competencies enabling them to work in a multidisciplinary team in a patient-centered healthcare system [4, 5]. This also challenges educationalists because the question can be raised how to design medical education which results in specialized knowledge and general competencies at the same time. The University Medical Center Groningen (UMCG) designed a competency-based curriculum called G2020 that on the one hand aims to train independent and excellent future specialists who acquire professional competencies and specialist knowledge, and on the other hand, have an early focus already in the bachelor phase on fields with expected future high demands. G2020 contains both shared content (same in four parallel programs) and specific content for four parallel programs (different in four parallel programs).

Competency-based medical education requires an outcome-based medical curriculum that specifies the competence requirements of students for good performance within the health system and assesses their achievements and shortcomings [4, 6–8]. Since undergraduate medical education primarily does not take place in the authentic clinical workplace, G2020 is designed in such a way that it comes as close as possible to the authentic clinical workplace. One such strategy is Problem Based Learning (PBL). Previous studies demonstrated that PBL helps students’ development of the seven CanMEDS competencies [9–13]. The CanMEDS framework is the most commonly used and integrated model to describe seven key competencies of physicians in both North America and Europe [14, 15]. It differentiates one critical integrating role the Medical Expert and six intrinsic roles, such as Communicator, Collaborator, Leader, Health Advocate, Scholar, and Professional [15].

Another approach is creating learning communities (LCs). LCs help students who share common academic goals and attitudes to meet regularly to collaborate on classwork which is known to benefit both their experience sharing, peer relationships, and professional competencies development [16–19]. LCs are adopted by many medical schools but only a few considered thematic learning communities (TLCs) [20]. TLCs are learning communities with their ‘own’ specific theme, profile, topic, context, learning process (task), and teaching faculty. It aims to deepen and expand students’ understanding of their preferred future career and avoid mismatching between students’ preferences and public health needs. It helps physicians’ training by using relevant content or discussing the same content under different themes, promoting students’ acquisition of specific knowledge and competencies. In G2020, we have four TLCs, each has its own theme reflecting the type of physician that the different TLCs intend to train. Considering the globalization of the curriculum and the number of international students, two TLCs are taught in Dutch containing domestic students only and another two were taught in English containing both domestic and international students. In our previous study, we focused on the assessment results of three competencies (collaboration, leadership, and professionalism) that are trained on the same content in four parallel programs to avoid the direct influence of the differences in the programs of the TLCs [21]. From that study, we learned that diverse groups with mixtures of international and domestic students achieved better results. In this study, we extend the previous study by comparing all seven competencies that are trained on different contents in four parallel programs. Besides, we also added students’ knowledge assessment results to have a more comprehensive understanding of students’ academic performance in G2020 and the effect of four parallel TLCs on students’ learning outcomes. By doing so, we explore if students’ learning outcomes reflect the characteristics of TLCs.

Thus, G2020 follows an innovative approach in medical education to train physicians in core professional competencies by organizing CBME in combination with TLCs and PBL cycles. This study will provide an overview of G2020 and compare the assessment results of students’ performances in this curriculum between four TLCs to answer the main research question: is it possible to acquire a similar level of learning outcomes in four different parallel programs within the same curriculum that is thought in two different languages?

## Method

### Educational background

#### Curriculum design of G2020

In the Netherlands, undergraduate medical education takes six years, divided into a bachelor phase (the first three years) and a master phase (the next three years) [22]. One academic year has two semesters and each semester has two blocks of ten weeks each. In G2020, the first bachelor year focuses on ten themes concerning various causes of disease. These themes address the major medical conditions of each discipline. The second bachelor year focuses on the major medical conditions relating to internal medicine, surgery and neurology. The third bachelor year mostly focuses on psychiatry, obstetrics/gynaecology and paediatrics. The first master year consists of four internships. Each internship lasts ten weeks, divided into a five-week period in which students train all necessary skills for practice and a five-week internship in the clinic. In this way, they alternate skills training and practice in the clinic four times during the first year. The second master year consists of 10 to 12 internships in different disciplines in the hospital. The third master year consists of two parts: an internship of 20 weeks in one discipline and a research project which will also last 20 weeks. This study describes the curriculum design of the bachelor phase in detail and compares the performance of students in the first two bachelor years between TLCs.

#### Four thematic LCs

The G2020 program is built upon four thematic learning communities for the bachelor phase: Sustainable Care (SC), Intramural Care (IC), Global Health (GH), and Molecular Medicine (MM). The selection of these themes is based on the connection to the expected development of healthcare, the UMCG research focuses, and the personal interest of future physicians. In response to the challenges of the health care professions, future physicians need to be able to coordinate long-term care, translate technological and fundamental scientific developments into affordable clinical care, maintain good relationships with other colleagues in a multidisciplinary team, and put healthcare issues into a broader perspective [4, 23]. TLC Sustainable Care (SC) is aiming to train students to optimize longitudinal care for patients and groups of patients, and focus on the relevant medical, social, ethical, and financial implications. It provides students necessary knowledge and skills to coordinate long-term healthcare for the individual patient and for groups of patients, collaborating with other healthcare professionals. Academic training in TLC SC focuses on first-line healthcare, epidemiology, lifestyle, and prevention. TLC Intramural Care (IC) trains students to translate thorough knowledge of diseases into quality medical care for individual patients and clinical research concerning groups of patients. It also focuses on working in multidisciplinary teams and peer assessment. TLC IC focuses on clinical and translational research in academic training. TLC Global Health (GH) aims to train students to acquire a global vision of healthcare beyond disciplinary boundaries [24]. It will give future physicians the ability to understand implications of global health both in daily healthcare practice and when entering the international medical job market. Academic training in TLC GH focuses on healthcare systems, indicators and disease in relation to political, social and economic factors. TLC Molecular Medicine (MM) trains students to use the latest technology to explore the molecular basis of diseases and the related diagnostic and therapeutic possibilities. It enables future physicians to participate in innovative fundamental biomedical or technological research. TLC MM focuses on translational and fundamental research. In addition, the TLC SC and TLC IC are taught in Dutch containing domestic students only and TLC GH and TLC MM are taught in English containing both domestic and international students.

TLCs allow students to acquire generic as well as specific competencies by immersing themselves in several different medical issues. This bachelor program aims for early distinguishing and focusing on future specialized physicians that on the other hand acquire the same basic competencies so that they are able to work efficiently in different health care systems.

Each year 410 students enter the G2020 program and they are assigned to four TLCs based on their own academic interests and language preferences as expressed during the selection system before the start of the study. Students stay in the same TLC for the entire bachelor phase of their study. After acquiring their bachelor’s diploma, they all start the same master’s program and there is no distinction anymore between students coming from different TLCs.

#### Structure of bachelor phase of G2020

**Fig. 1:**
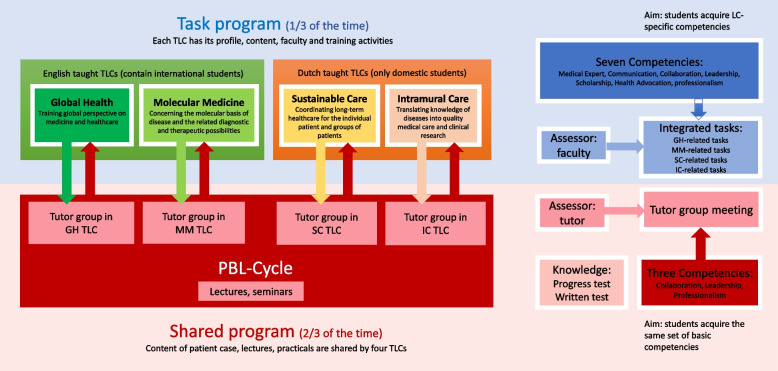
Curriculum design of G2020

During the entire bachelor phase, the G2020 curriculum contains a shared program and a TLC-specific task program which consists of a coherent body of integrated tasks (see Fig. 1). The shared program takes up about two-thirds of the time while the TLC-specific task program takes one-third of the time. Competence development takes place throughout the curriculum, both in the shared program and in the TLC-specific tasks.

The shared program is similar for the four TLCs but taught in two languages. In the TLCs, PBL cycles with tutor groups form the starting point of learning supported by lectures, seminars, and practical sessions. Students are divided into several tutor groups for collaborative learning. Ten students from the same TLC form a tutor group with a master student as a tutor. The tutor works as an observer, guide, and assessor. The tutor observes students’ behavior and guides students to come up with questions or deal with students’ problems during the meeting. At the end of each block, the tutor gives students a score of their competencies based on their performance in the group meetings.

The Competency development program (TLC-specific task program) in the four TLCs differs from each other. Each TLC has its own profile, content, task activities, learning materials, and assessments. The task program trains knowledge relating to the TLC profile or increases medical knowledge in general. It also focuses on the important aspects of academization: practicing science and the academic attitude. Students in the task program need to complete several TLC-specific tasks every semester. Each task trains and assesses two or three competencies. The duration of each task varies. Through the task programs, students acquire all seven competencies. The task program contains science groups and coaching groups. The science group introduces students to specific fields of science and develops students’ specific skills. The coaching group introduces the physicians’ professional working environment to students in the early stage of the degree program, completes specific tasks, and then discusses the experiences to cultivate the students’ academic attitude. In the coaching group, students can learn from experience, reflection, and intervention. The consultation is also taught together with the coaching group in order to maintain an understanding of the medical background and gain practical experience at an early stage.

### Participants

In this study, we analyzed the learning outcomes of students in the first two bachelor years of the University Medical Center of Groningen (UMCG) in the Netherlands. Here, we used the results of three cohorts of students, 1215 students (68% female, 84% domestic students) in total, namely the 2014–2015 (BA1415), the 2015–2016 (BA1516), and the 2016–2017 (BA1617) cohort (see Table 1).

**Table 1:** Description of participants of all cohorts

Cohorts	Language	LC	Student Number	Sex	Age	Country of origin
				Female (%)	(mean)	Domestic (%)
BA1415	Dutch	SC	75	49 (65.3)	19.35	75 (100)
		IC	121	85 (70.2)	18.88	120 (99.2)
	English	GH	105	69 (65.7)	19.54	79 (75.2)
		MM	92	56 (60.9)	19.21	69 (75.0)
		**Total**	**393**	**259 (65.9)**	**19.22**	**343 (87.3)**
BA1516	Dutch	SC	98	78 (79.6)	18.72	96 (98.0)
		IC	144	102 (70.8)	18.94	144 (100)
	English	GH	86	63 (73.3)	19.64	45 (52.3)
		MM	74	48 (64.9)	19.30	50 (67.6)
		**Total**	**402**	**291 (72.4)**	**19.10**	**335 (83.3)**
BA1617	Dutch	SC	73	51 (69.9)	19.01	72 (98.6)
		IC	157	109 (69.4)	18.76	156 (99.4)
	English	GH	89	60 (67.4)	19.25	53 (59.6)
		MM	101	59 (58.4)	19.56	65 (64.4)
		**Total**	**420**	**279 (66.4)**	**19.10**	**346 (82.4)**
Total			1215	829 (68.2)	19.14	1024 (84.3)

*SC* = Sustainable Care, *IC* = Intramural Care, *GH* = Global Health, *MM* = Molecular Medicine

### Measurements

We compared students’ learning outcomes among four TLCs to investigate the variations of students’ obtained learning outcomes. To compare students’ performance between TLCs, we used the following students’ assessment results:

#### Knowledge

Students’ knowledge performances were assessed by the results of written tests and progress tests. The written test is an internal program-dependent test (designed by faculty and related to study material used in class) and assesses students’ medical knowledge. Every semester has 4 or 5 written test moments. The results of these written tests are combined as a one-semester test with scores ranging from 0 to 10 (higher scores indicate better performance). The passing score is 5.5. English taught TLCs and Dutch taught TLCs use the same questions but in different languages.

The progress test is a medical knowledge test used by different universities in parallel with a long history in the Netherlands [25]. It is an external, curriculum-independent test to be used as a formative assessment monitoring knowledge growth [26]. It consists of four tests every academic year and has a summative result at the end of each year.

#### Competencies

**Fig. 2:**
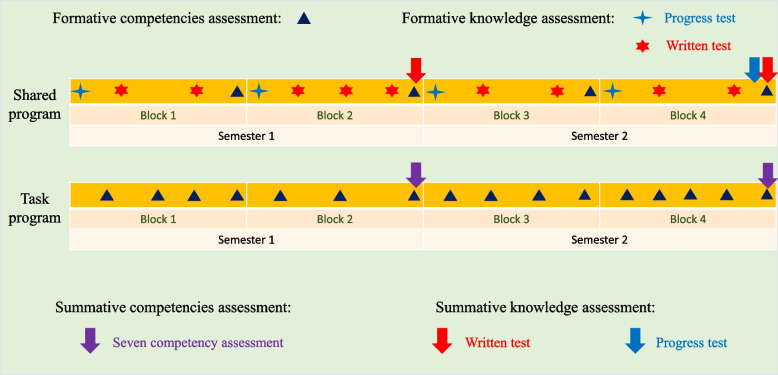
The timeline of assessments in G2020

Competencies assessment was obtained by observing students’ performance by faculty. The assessment program of competencies (see Fig. 2) puts emphasis on many formative evaluations with a focus on feedback for the students’ behavior and to present the level of students’ knowledge application [14]. Competencies are assessed by programmatic assessment with many formative evaluations resulting in a final summative decision [27]. The competencies assessments in G2020 use a three-scale score: Fast-on-track (FOT; i.e., performing excellent), On-track (OT; i.e., performing at an average level), and Not-on-track (NOT; i.e., failing) for the formative assessments.

In the shared program, four TLCs were assessed at the same timepoint by tutors. Tutors assess students’ three competencies (Collaboration, leadership, and Professionalism) at the end of each block. In total, those three competencies are assessed eight times in the first two years (see Fig. 2). We have reported those results earlier and will not repeat them here [21].

In this study, we focused on students’ competencies assessment results in task programs, which differ between the four TLCs. In the task program, students’ seven competencies are assessed by different assessors at the end of each task, such as long-term coaches, occasional experts, peers, or through self-evaluation. In the task program, each task assesses two to three competencies. Every block contains three to five tasks. At the end of each semester, the summative result of students’ competencies assessment will be made depending on the number of NOT, OT, and FOT evaluations in both shared program and task program. An overview of the different tasks and which competencies are assessed in which task is shown in Additional file 1.

### Data collection and data analysis

This study collected students’ knowledge and competencies assessment results, as well as their background information (TLC, age, sex, nationality) from several databases from the administration office of the UMCG Medical Faculty after their first two years of study, and all personal data were anonymized before use in our analysis. This study collected the first and second-year assessments results for all cohorts. The study was approved by the Ethical Review Board of the Netherlands Association of Medical Education (NVMO), dossier number 2019.4.8.

This study used the cumulative deviation method to present the result of students’ progress tests. It is a widely used method to analyze progress test results [26, 28, 29]. This method first compares the mean score of students’ progress tests between TLCs, then it shows the deviation of each TLC from the overall mean. Positive scores reflect performances higher than average of the other TLCs and negative values reflect performances below the average of the other TLCs. Then the method calculated cumulative deviation scores to provide a clearer view of the systematic differences between the four TLCs.

Besides, this study also used the average score of the written tests per semester to present students’ knowledge assessment results. Since the written test scores were not normally distributed, we conducted the Kruskal–Wallis H test to explore if students’ average written test score differs across TLCs per semester. Students’ competencies assessment results in the task program are presented by the percentage of FOT, OT, and NOT per semester per competency. Descriptive statistics are used to present all the results of the students’ competencies.

## Results

To test whether students can acquire similar learning outcomes (both seven competencies performance and knowledge development) in four TLCs, this study compared students’ seven competencies assessment results in the task program among four TLCs, and used students’ written test scores and progress test scores to present the differences of students’ knowledge development across four TLCs.

### Students’ knowledge assessments

**Fig. 3:**
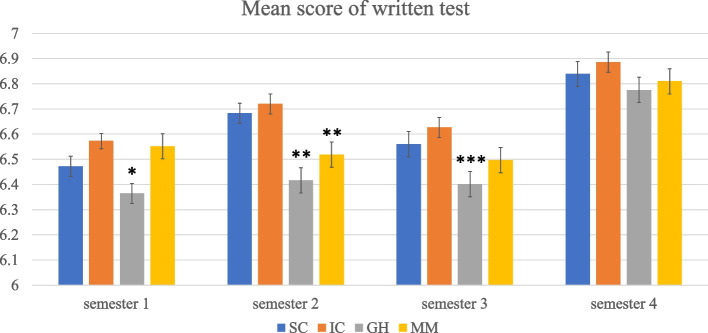
Mean score of written test score over time. The asterisk presents the significant difference between LCs. SC = Sustainable Care, IC = Intramural Care, GH = Global Health, MM = Molecular Medicine. *: the mean scores of TLC GH are significantly lower than the other three TLCs in semester 1 (*H* (3) = 17.672, *p* = . 001). **: the mean scores of TLC GH and TLC MM (two English TLCs) scores are significantly lower than TLC SC and TLC IC (two Dutch TLCs) in semester 2 (*H* (3) = 27.742, *p* < .001). ***: the mean scores of TLC GH are significantly lower than TLC SC and TLC IC (two Dutch TLCs) in semester 3 (*H* (3) = 11.626, *p* = .009)

We used the average score of the written tests per semester to present the result of the student’s knowledge assessment. Concerning the passing rates, the majority of students passed all assessments (see Table 2). Besides, students’ written test score became higher during the first two years. Figure 3 shows differences in students’ written test scores between four TLCs. English TLCs showed lower scores than Dutch TLCs at the beginning, especially TLC GH, but showed similar scores with Dutch TLCs at the later stage. In the first semester, TLC GH showed a significantly lower score than the other three TLCs (*H* (3) = 17.672, p = . 001). The mean score of TLC GH was 6.36 (*SD* = 0.04) while other three TLCs were 6.47 (*SD* = 0.04), 6.57 (*SD* = 0.03), and 6.55 (*SD* = 0.05) respectively. In the second semester, a significant difference is seen between English and Dutch TLCs (*H* (3) = 27.742, *p* < 0.001). The mean score of two English TLCs were 6.42 (*SD* = 0.05) and 6.52 (*SD* = 0.05) respectively while two Dutch TLCs were 6.68 (*SD* = 0.04), 6.72 (*SD* = 0.04) respectively. In the third semester, the TLC GH showed a significantly lower score than the two Dutch TLCs (*H* (3) = 11.626, *p* = 0.009). The mean score of TLC GH was 6.40 (*SD* = 0.05) while two Dutch TLCs were 6.56 (*SD* = 0.05), 6.63 (*SD* = 0.04) respectively. In the fourth semester, there was no significant difference between TLCs. The mean score of four TLCs were 6.85 (*SD* = 0.05), 6.87 (*SD* = 0.04), 6.76 (*SD* = 0.05), and 6.81 (*SD* = 0.05) respectively.

**Table 2:** Passing rates for the written tests

TLC	Semester 1	Semester 2	Semester 3	Semester 4
SC	91.83%	94.51%	93.28%	91.29%
IC	95.08%	95.99%	94.29%	93.70%
GH	87.42%	85.03%	91.04%	89.57%
MM	91.07%	87.05%	90.11%	89.27%

*SC* = Sustainable Care, *IC* = Intramural Care, *GH* = Global Health, *MM* = Molecular Medicine

**Fig. 4:**
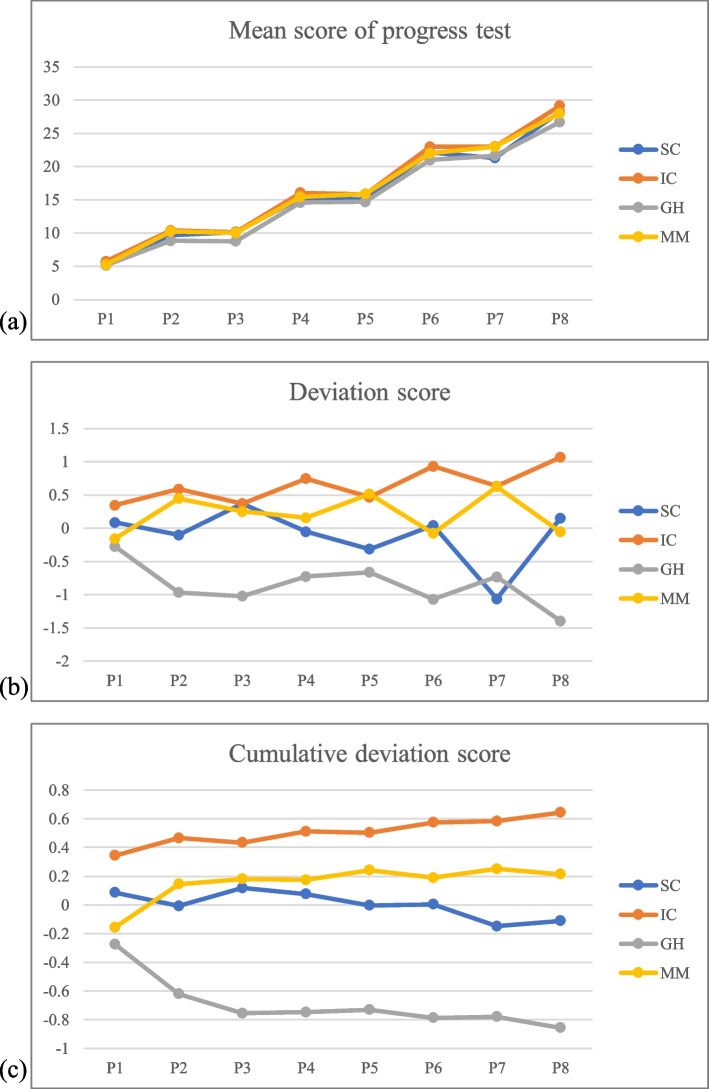
Score for the three cohorts on progress test in the first two academic years

The differences in students’ knowledge performances between the four TLCs were also revealed by progress tests (see Fig. 4). The passing rate of the progress test is 97.7% for the first bachelor year and 99.6% for the second bachelor year. Most of the students have passed the progress test though they were in different TLCs, the results are similar with students’ written test results. Figure 4a shows the growth of students’ medical knowledge over time, as the mean raised from 5 to 28. Most of the time TLC IC showed the highest mean score when comparing it with other TLCs while the TLC GH showed the lowest mean score. Figure 4b illustrates each TLCs deviation from the overall mean. TLC GH performed lower than other TLCs most of the time. As Fig. 4c shows, the last assessment within one academic year (P4, P8) reflects the average overall performance of a TLC across that period. The knowledge assessment performance of TLC GH was relatively decreased over time compared to other TLCs.

In general, TLC GH showed the lowest performance in both two knowledge assessments and TLC IC performed best most of the time.

### Students’ competencies assessments

The students’ performances in the task program depend on the overall competencies’ performances. In total, only seven students failed in the first semester, six students failed in the second semester and three students failed in the fourth semester. The percentage was always lower than 1%. The percentage of NOT for all seven competencies varied from 1.91% to 7.51%. Most of them were lower than 5%. This means not only that the final passing rate was high, but also that the passing rates of each assessment were high as well. Most of the students passed all assessments. It reflects that students are able to acquire the necessary level of competencies when they were in different TLCs although with a few differences.

**Fig. 5:**
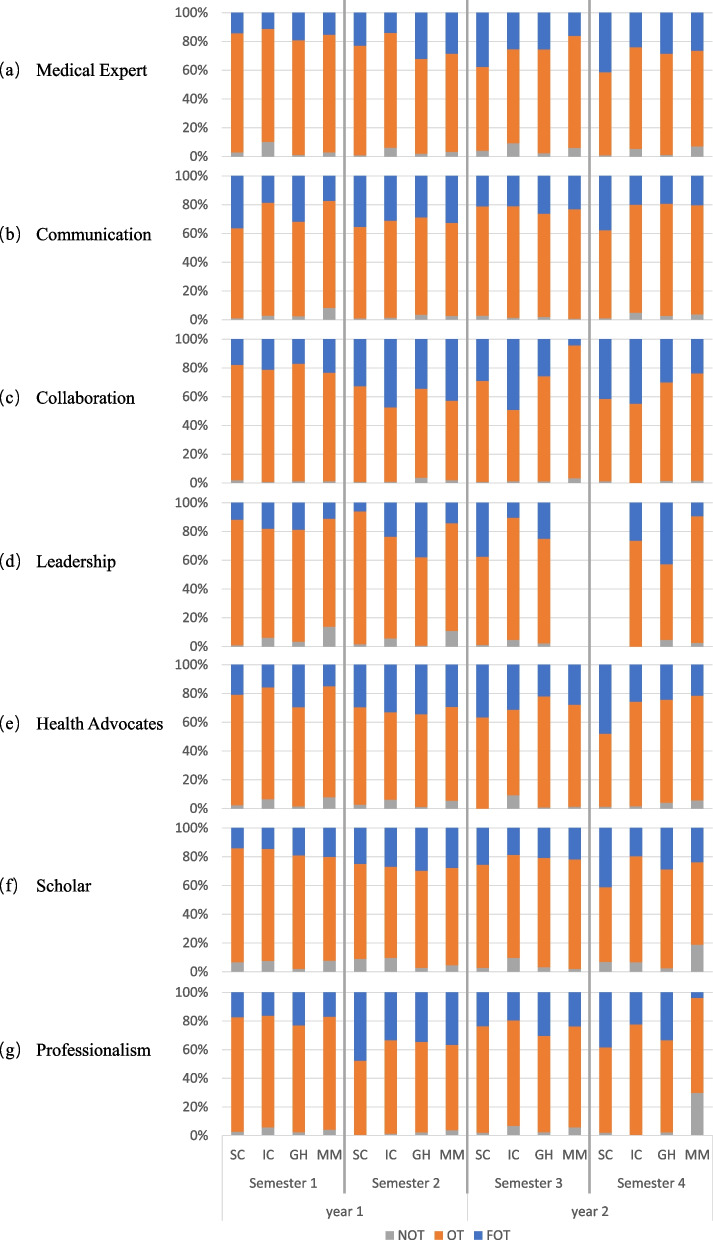
Students competencies assessment results per competency (the percentage of NOT, OT and FOT). FOT = Fast-on-track, OT = On-track, NOT = Not-on-track. SC = Sustainable Care, IC = Intramural Care, GH = Global Health, MM = Molecular Medicine

Table 3 shows that students’ competencies performance became higher at the end of the second year in all TLCs, which is similar to what is observed earlier in the competencies result of the shared program [21]. The percentages of FOT for all competencies together varied from 16.21% to 41.63% and the percentages of FOT became higher at the end of the second year although there were some variations over time. However, it is quite difficult to compare directly students’ competencies performances in the task program between TLCs since there are so many variables in the diverse task program design, such as varying time points of assessments and different types of assessors. In general, TLC SC and TLC GH had relatively higher percentages of FOT and relatively lower percentages of NOT than TLC IC and TLC MM (see Fig. 5).

**Table 3:** Students’ competency assessment results per LC per semester

**LC**	**Competency**		**Semester 1**	**Semester 2**	**Semester 3**	**Semester 4**
			N (%)	N (%)	N (%)	N (%)
**SC**	Medical expert	FOT	124 (14.14)	170 (22.85)	181 (37.71)	125 (41.25)
		OT	728 (83.01)	567 (76.21)	279 (58.13)	175 (57.76)
		NOT	25 (2.85)	7 (0.94)	20 (4.17)	3 (0.99)
	Communication	FOT	64 (36.36)	328 (35.38)	51 (21.16)	269 (37.78)
		OT	110 (62.50)	590 (63.65)	183 (75.93)	435 (61.10)
		NOT	2 (1.14)	9 (0.97)	7 (2.90)	8 (1.12)
	Collaboration	FOT	71 (17.93)	215 (32.67)	70 (29.05)	99 (41.60)
		OT	317 (80.05)	438 (66.57)	169 (70.12)	136 (57.14)
		NOT	8 (2.02)	5 (0.76)	2 (0.83)	3 (1.26)
	Leadership	FOT	47 (11.72)	11 (6.01)	180 (37.50)	
		OT	350 (87.28)	169 (92.35)	294 (61.25)	
		NOT	4 (1.00)	3 (1.64)	6 (1.25)	
	Health advocate	FOT	190 (20.86)	194 (29.44)	88 (36.67)	371 (47.87)
		OT	699 (76.73)	447 (67.83)	152 (63.33)	394 (50.84)
		NOT	22 (2.41)	18 (2.73)	0 (0.00)	10 (1.29)
	Scholar	FOT	120 (14.05)	143 (24.96)	135 (25.52)	150 (41.10)
		OT	677 (79.27)	379 (66.14)	380 (71.83)	190 (52.05)
		NOT	57 (6.67)	51 (8.90)	14 (2.65)	25 (6.85)
	Professionalism	FOT	120 (17.27)	278 (47.68)	68 (23.53)	207 (38.33)
		OT	556 (80.00)	302 (51.80)	215 (74.39)	322 (59.63)
		NOT	19 (2.73)	3 (0.51)	6 (2.08)	11 (2.04)
	**Total (SC)**	**FOT**	**736 (17.08)**	**1339 (30.95)**	**773 (30.92)**	**1221 (41.63)**
		**OT**	**3437 (79.74)**	**2892 (66.84)**	**1672 (66.88)**	**1652 (56.32)**
		**NOT**	**137 (3.18)**	**96 (2.22)**	**55 (2.20)**	**60 (2.05)**
**IC**	Medical expert	FOT	133 (11.15)	183 (14.00)	306 (25.25)	204 (23.89)
		OT	938 (78.63)	1044 (79.88)	794 (65.51)	605 (70.84)
		NOT	122 (10.23)	80 (6.12)	112 (9.24)	45 (5.27)
	Communication	FOT	253 (18.56)	374 (31.09)	127 (21.06)	244 (19.98)
		OT	1071 (78.58)	812 (67.50)	467 (77.45)	917 (75.10)
		NOT	39 (2.86)	17 (1.41)	9 (1.49)	60 (4.91)
	Collaboration	FOT	156 (21.17)	570 (47.46)	344 (49.14)	275 (44.79)
		OT	576 (78.15)	620 (51.62)	347 (49.57)	339 (55.21)
		NOT	5 (0.68)	11 (0.92)	9 (1.29)	0 (0.00)
	Leadership	FOT	162 (18.00)	188 (23.50)	38 (10.41)	97 (26.43)
		OT	682 (75.78)	566 (70.75)	310 (84.93)	270 (73.57)
		NOT	56 (6.22)	46 (5.75)	17 (4.66)	0 (0.00)
	Health advocate	FOT	146 (15.77)	440 (32.93)	231 (31.30)	218 (25.62)
		OT	719 (77.65)	814 (60.93)	439 (59.49)	619 (72.74)
		NOT	61 (6.59)	82 (6.14)	68 (9.21)	14 (1.65)
	Scholar	FOT	175 (14.53)	246 (26.91)	91 (18.69)	143 (19.59)
		OT	937 (77.82)	580 (63.46)	349 (71.66)	538 (73.70)
		NOT	92 (7.64)	88 (9.63)	47 (9.65)	49 (6.71)
	Professionalism	FOT	125 (16.19)	301 (33.26)	71 (19.45)	55 (22.36)
		OT	603 (78.11)	591 (65.30)	269 (73.70)	191 (77.64)
		NOT	44 (5.70)	13 (1.44)	25 (6.85)	0 (0.00)
	**Total (IC)**	**FOT**	**1150 (16.21)**	**2302 (30.03)**	**1208 (27.02)**	**1236 (25.31)**
		**OT**	**5526 (77.89)**	**5027 (65.58)**	**2975 (66.65)**	**3479 (71.25)**
		**NOT**	**419 (5.91)**	**337 (4.40)**	**287 (6.42)**	**168 (3.44)**
**GH**	Medical expert	FOT	194 (19.04)	313 (32.14)	288 (25.42)	251 (28.39)
		OT	813 (79.78)	641 (65.81)	819 (72.29)	623 (70.48)
		NOT	12 (1.18)	20 (2.05)	26 (2.29)	10 (1.13)
	Communication	FOT	232 (31.74)	323 (28.87)	162 (26.17)	171 (19.32)
		OT	481 (65.80)	756 (67.56)	445 (71.89)	690 (77.97)
		NOT	18 (2.46)	40 (3.57)	12 (1.94)	24 (2.71)
	Collaboration	FOT	97 (16.93)	318 (34.30)	169 (25.80)	148 (30.08)
		OT	469 (81.85)	574 (61.92)	479 (73.13)	337 (68.50)
		NOT	7 (1.22)	35 (3.78)	7 (1.07)	7 (1.42)
	Leadership	FOT	98 (18.77)	106 (37.72)	56 (25.00)	156 (42.74)
		OT	409 (78.35)	173 (61.57)	163 (72.77)	192 (52.60)
		NOT	18 (3.45)	2 (0.71)	5 (2.23)	17 (4.66)
	Health advocate	FOT	287 (29.44)	311 (34.36)	169 (22.03)	188 (24.29)
		OT	673 (69.03)	583 (64.62)	592 (77.18)	554 (71.58)
		NOT	15 (1.54)	11 (1.22)	6 (0.78)	32 (4.13)
	Scholar	FOT	208 (19.01)	214 (29.68)	175 (20.88)	224 (28.87)
		OT	865 (79.07)	488 (67.68)	636 (75.89)	532 (68.56)
		NOT	21 (1.92)	19 (2.64)	27 (3.22)	20 (2.58)
	Professionalism	FOT	113 (23.01)	284 (34.51)	110 (30.39)	150 (33.26)
		OT	366 (74.54)	521 (63.30)	243 (67.13)	291 (64.52)
		NOT	12 (2.44)	18 (2.19)	9 (2.49)	10 (2.22)
	**Total (GH)**	**FOT**	**1229 (22.74)**	**1869 (32.50)**	**1129 (24.55)**	**1288 (27.84)**
		**OT**	**4076 (75.41)**	**3736 (64.97)**	**3377 (73.44)**	**3219 (69.57)**
		**NOT**	**103 (1.91)**	**145 (2.52)**	**92 (2.00)**	**120 (2.59)**
**MM**	Medical expert	FOT	117 (15.16)	206 (28.37)	114 (16.03)	162 (26.34)
		OT	633 (81.99)	497 (68.46)	554 (77.92)	410 (66.67)
		NOT	22 (2.85)	23 (3.17)	43 (6.05)	43 (6.99)
	Communication	FOT	104 (17.36)	232 (32.58)	191 (23.12)	125 (20.29)
		OT	445 (74.29)	461 (64.75)	628 (76.03)	468 (75.97)
		NOT	50 (8.35)	19 (2.67)	7 (0.85)	23 (3.73)
	Collaboration	FOT	172 (23.21)	170 (42.71)	9 (4.39)	70 (23.73)
		OT	559 (75.44)	220 (55.28)	189 (92.20)	220 (74.58)
		NOT	10 (1.35)	8 (2.01)	7 (3.41)	5 (1.69)
	Leadership	FOT	38 (11.14)	42 (14.24)		19 (9.31)
		OT	260 (76.25)	221 (74.92)		180 (88.24)
		NOT	48 (14.08)	32 (10.85)		5 (2.45)
	Health advocate	FOT	108 (14.90)	194 (29.31)	172 (27.83)	176 (21.54)
		OT	560 (77.24)	432 (65.26)	438 (70.87)	595 (72.83)
		NOT	57 (7.86)	36 (5.44)	8 (1.29)	46 (5.63)
	Scholar	FOT	198 (20.16)	236 (27.70)	136 (21.90)	49 (23.79)
		OT	708 (72.10)	576 (67.61)	473 (76.17)	118 (57.28)
		NOT	76 (7.74)	40 (4.69)	12 (1.93)	39 (18.93)
	Professionalism	FOT	41 (16.87)	167 (36.62)	98 (23.67)	8 (3.92)
		OT	192 (79.01)	272 (59.65)	292 (70.53)	135 (66.18)
		NOT	10 (4.12)	17 (3.73)	24 (5.80)	61 (29.90)
	**Total (MM)**	**FOT**	**778 (17.67)**	**1247 (30.41)**	**720 (21.21)**	**609 (20.60)**
		**OT**	**3357 (76.24)**	**2679 (65.33)**	**2574 (75.82)**	**2126 (71.90)**
		**NOT**	**273 (6.20)**	**175 (4.27)**	**101 (2.97)**	**222 (7.51)**

*FOT* = Fast-on-track, *OT* = On-track, *NOT* = Not-on-track, *SC* = Sustainable Care, *IC* = Intramural Care, *GH* = Global Health, *MM* = Molecular Medicine

Due to the differences in task programs between the four TLCs, students’ competencies performance differs across TLCs every semester (see Table 3 and Additional file 1). Overall, 66 to 73% of students get OT and around 22 to 32% of students get FOT in all competencies and only 1 to 5% of students failed. Sometimes, however, the percentage of NOT was even higher than 20% in some competencies. TLC MM showed a high percentage of NOT in Scholar and Professionalism. Besides, different TLCs showed relatively high performance in different competencies. TLC IC showed a relatively high percentage of FOT in Collaboration. TLC SC showed a relatively high percentage of FOT in Medical Experts and Communication. TLC GH showed a relatively high percentage of FOT in Leadership. In addition, we found that some competencies were easier to get high scores in performance assessment than other competencies and some are more difficult. Collaboration showed the highest percentage of FOT and the lowest percentage of NOT. While Leadership shows the lowest percentage of FOT and Scholar shows the highest percentage of NOT. Leadership was not assessed in the third semester in the TLC MM as well as in the fourth semester for TLC SC. Every semester the performances of different competencies fluctuate, but TLC SC performed best in the fourth semester for almost all competencies.

## Discussion

The innovative curriculum G2020 combines TLCs and PBL with CBME in order to train students to acquire a similar level of core professional competencies but with different specific knowledge areas and competencies. This study compared students’ knowledge and competencies assessment results in the first two bachelor years between four TLCs with different curriculum design indicates that it is possible to train students in different parallel programs within the same curriculum while reaching a similar level of learning outcomes.

Most of the students passed all kinds of assessments of competencies (students who got Fast-on-track and On-track), and the rate of failure was quite low (lower than 5%). It indicates that even though students were in different TLCs, most students reached the basic requirements of competencies. It is possible for students to obtain the required learning outcomes in different TLCs, but with variations. We observed that TLC SC and TLC GH had relatively higher percentages of FOT and relatively lower percentages of NOT than TLC IC and TLC MM. Otherwise, we found TC IC and TLC MM outperformed TLC SC and TLC GH on the progress test. This difference might be explained by the fact that TLCs IC and MM focus more on knowledge development. The other side of these assessment results is that those TLCs show relatively lower competencies performance than TLC SC and TLC GH. In contrast to the previous study where we showed that the two English taught TLCs showed higher competencies assessment results than two Dutch taught TLCs [21], we now found that the Dutch TLCs had better written test scores than two English TLCs in the first year. There seems to be a clear trade-off between the focus on competencies or knowledge development, especially in the first year. We did not observe differences in written test scores between the four LCs at the end of the second year, suggesting that this effect is mostly seen in the early phase where students still need to adapt to our curriculum.

In addition, due to the diversity of the TLC task program, some students may feel unfair of their assessments. Consistent with Misbah et al., TLCs that are more focused on competencies development have consequently less time for knowledge development [30]. Students who tend to focus on written tests may feel they are unfairly treated when the TLC program is less focused on knowledge development. The workload of the task program also differs on account of the differences in the TLC tasks. Some TLCs have a higher workload in the task program than others. When students have a high workload in the task program they have less time for the shared program. For instance, TLC GH more focuses on competencies development by introducing tasks that require a high time investment in the TLC task program so students in TLC GH have less time on the shared program resulting in lower knowledge assessment results than other TLCs. This is reflected in the lower passing rates of the written tests for the TLC GH in the first year. But this does not seem to be the only explanation since the TLC MM also has lower passing rates in the second semester of the first year. This suggests that also language differences could be related to the observed differences. Although the questions in the written test and the progress test are translated by a professional translation service, it is still possible that some bias was introduced by the translation [31, 32]. These differences were not seen in the second year, suggesting that the students seem to adapt to the system.

In the shared program, tutors were assessors of students’ competencies and some of the results were based on their subjective evaluations [33]. To avoid bias, we changed tutors every half-semester and randomly distributed them across the tutor groups and across different TLCs to decrease the unfairness caused by differences in capacities of the tutors. However, we noticed the importance of tutors, not only for assessment but also for tutor group activities themselves [33–35]. Some tutors may feel more responsible and guide students better than other tutors. Although the tutor group has a weekly leader for group activities, many students may be incapable to be good leaders at the beginning. If so, students in tutor groups hardly make full use of the meeting time and have fewer in-depth discussions. They need guidance and to learn from their tutors. If a tutor gave more guidance for students concerning their group collaboration and group discussion, students may get better learning outcomes. Thus, when faculty trains tutors before the beginning of each semester, especially tutors for the first-year students, they need to pay more attention to how tutors can assist students to organize the group meeting.

### Strengths and limitations of the study, further research and implications for practice

One strength of this study is that we explored to what extent students’ competencies and knowledge assessment results differ due to the difference in curriculum design in task programs, which provides a practical experience for curriculum designers who would like to attempt diverse curriculum design. Furthermore, we compared students’ academic performance over the first two years which provides a clear long observation result of the effect of diverse curriculum design on medical undergraduate students’ academic performance. Additionally, we compared the results of this study with our previous study and presented differences in the impact of the same curriculum design and the diverse curriculum design on students’ academic performance.

However, the deficiencies of the curriculum design in this study are also worth noting. Although the three-scale score is easy to mark scores for students, we should be careful with interpreting the result, because the scoring of the competencies is less standardized than the written test score and the progress test. The type of assessors and assessment times also differed between TLCs. Therefore, it is difficult to compare all assessment results for competencies across TLCs since there are so many differences in the diverse task program design, such as the different number of tasks per competency in each of the TLCs.

Thus, the curriculum design needs to be improved to find the balance between comparability of assessment and diversity of curriculum design in the future. Therefore, future studies could consider the ten-scale score, which may be preferable to the three-scale score for statistical evaluation purposes although this may make it more difficult for assessors to grade students’ performances.

Since we found a trade-off between the focus on competencies or knowledge development, curriculum designers need to learn from the observed differences in our study when they attempt to use different parallel designs for different groups of students. Curriculum designers need to carefully balance between knowledge and competencies development in the characteristics of the TLC-specific programs.

## Conclusion

To sum up, this study provides evidence that early focus on future specialization in different TLCs is possible within the same CBME program and it offers a new way of curriculum design. There are no serious differences found in knowledge and competence development across TLC, and thus being part of one TLC will not hamper the student development compared with the students in other TLCs. The variation in obtaining learning outcomes is acceptable and does not cause any study delay. Students are all ready to follow the master’s education that is equal for all students independent from the TLC where they did their bachelor’s study. Since the implementation of CBME is always iterative and dynamic by merging new theories and improving training programs constantly, we expect improved curriculum programs and more new curriculum designs based on CBME in the future. There may be an improvement of curriculum design by changing the scoring system for students’ competencies assessment. By increasing tutors’ influence in tutor groups we might provide better guidance to improve team collaboration. The final aim of the G2020 curriculum steering future physicians towards future career directions with an expected high demand by early exposure still needs to be validated by longitudinal research following the alumni for their medical career.

## Supplementary Information

**Additional file 1.** The overview of tasks and competencies assessment for the four thematic learning communities.

## Data Availability

The dataset used and analyzed during this study are available from the corresponding author upon reasonable request.

## Abbreviations

PBL: Problem-based learning
CBME: Competency-based medical education
LC: Learning Communities
TLC: Thematic Learning community
SC: Sustainable Care
IC: Intramural Care
GH: Global Health
MM: Molecular Medicine
FOT: Fast On Track
OT: On Track
NOT: Not On Track
