# Supplementary material for: Challenges to acquire similar learning outcomes across four parallel thematic learning communities in a medical undergraduate curriculum
Source: BMC Med Educ. 2023 May 18;23:349. doi: 10.1186/s12909-023-04341-x (PMC10193746; doi:10.1186/s12909-023-04341-x)
Supplement: Supplementary file 1 — Additional file 1. The overview of tasks and competencies assessment for the four thematic learning communities. [file 12909_2023_4341_MOESM1_ESM.docx]

**Additional file 1.** The overview of tasks and competencies assessment for the four thematic learning communities

| LC | | Task name | | MED | | COM | | COL | | LEA | | HA | | SCH | | PRO | |
| --- | --- | --- | --- | --- | --- | --- | --- | --- | --- | --- | --- | --- | --- | --- | --- | --- | --- |
| First year | | | | | | | | | | | | | | | | | |
| SC | | Task 1.1 - Downscreening, what do you want to know | |  | |  | |  | |  | | √ | |  | | √ | |
| SC | | Task 1.2 - Collaboration and integrated care for the patient with DM type II | |  | |  | | √ | | √ | |  | |  | |  | |
| SC | | Task 1.3a - Deep Vein Thrombosis | | √ | |  | |  | |  | |  | |  | | √ | |
| SC | | Task 1.3b - Patient-centered communication about cancer | |  | | √ | |  | |  | |  | | √ | |  | |
| SC | | Task 1.4 - Antibiotic Resistance | | √ | |  | |  | |  | | √ | |  | |  | |
| SC | | Task 1.5 - Occupational Allergy | |  | |  | |  | | √ | | √ | |  | |  | |
| SC | | Task 1.6 - Influencing cardiovascular risk | |  | |  | |  | |  | | √ | | √ | |  | |
| SC | | Task 1.7 - Pathophysiology of shock | | √ | |  | | √ | |  | |  | |  | |  | |
| SC | | Task 1.8 - The Doctor's most powerful weapon | | √ | | √ | |  | |  | |  | |  | |  | |
| SC | | Task 1.9 - Stress and coping | |  | | √ | |  | |  | |  | |  | | √ | |
| GH | | Task 1.1 - Child development - Down Syndrome - what is health | | √ | |  | |  | | √ | | √ | |  | |  | |
| GH | | Task 1.2 - Metabolism Diabetes Type 1 Evidence based medicine | |  | |  | |  | |  | | √ | | √ | |  | |
| GH | | Task 1.3a - Anaemia Worldwide | |  | |  | |  | |  | | √ | | √ | |  | |
| GH | | Task 1.3b - Basic Life Support | | √ | |  | | √ | |  | |  | |  | |  | |
| GH | | Task 1.3c - Consultation skills | | √ | | √ | |  | |  | |  | |  | |  | |
| GH | | Task 1.4 - Infectious Diseases - Assessment Treatment Global Epidemics | |  | | √ | |  | |  | |  | | √ | |  | |
| GH | | Task 1.5a - Living with HIV-AIDS | | √ | | √ | |  | |  | |  | |  | |  | |
| GH | | Task 1.5b - Global Health Symposium | |  | |  | | √ | |  | |  | |  | | √ | |
| GH | | Task 1.6 - End of life issue | | √ | | √ | |  | |  | |  | |  | | √ | |
| GH | | Task 1.7a - Road traffic injuries | | √ | |  | |  | |  | |  | | √ | |  | |
| GH | | Task 1.7b - Consultation skills | |  | | √ | | √ | |  | | √ | |  | |  | |
| GH | | Task 1.8b - Science internship | |  | |  | |  | |  | |  | | √ | | √ | |
| GH | | Task 1.9 - Pain relief in osteoarthritis | |  | |  | | √ | | √ | | √ | | √ | |  | |
| GH | | Task 1.10 - Occupational health | | √ | |  | |  | |  | | √ | |  | |  | |
| IC | | Task 1.1 - Get in shape | | √ | |  | |  | |  | |  | |  | | √ | |
| IC | | Task 1.2 - Treatment Advice for Patients with Diabetes Mellitus I | |  | | √ | |  | |  | | √ | | √ | |  | |
| IC | | Task 1.3a - Bloody Serious | | √ | | √ | |  | |  | |  | |  | |  | |
| IC | | Task 1.3b - Can we outsmart cancer | |  | |  | |  | | √ | |  | | √ | |  | |
| IC | | Task 1.4 - Diagnostics and prevention of infections in the hospital | |  | | √ | |  | |  | | √ | |  | |  | |
| IC | | Task 1.5 - Biological dMARDS - Sjogren's Syndrome | | √ | | √ | |  | |  | |  | | √ | |  | |
| IC | | Task 1.6 - Acute Medicine | | √ | | √ | |  | | √ | |  | |  | |  | |
| IC | | Task 1.7 - Orthopedics | |  | |  | |  | | √ | | √ | |  | |  | |
| IC | | Task 1.8 - Consulting | |  | | √ | |  | |  | | √ | |  | |  | |
| MM | | Task 1.1 - Antithrombotic therapy for liver disease | |  | |  | |  | | √ | |  | | √ | |  | |
| MM | | Task 1.2 - Genetic counselling | |  | | √ | |  | |  | | √ | |  | |  | |
| MM | | Task 1.3 - Immunological interactions | |  | |  | | √ | |  | |  | | √ | |  | |
| MM | | Task 1.4 - Informing a GP about a pathogen | | √ | |  | |  | |  | | √ | |  | |  | |
| MM | | Task 1.5 - Inventing therapies | | √ | |  | |  | |  | |  | | √ | |  | |
| MM | | Task 1.6 - Mechanisms of cancer | |  | |  | | √ | |  | |  | | √ | |  | |
| MM | | Task 1.7 - Biomaterials and regenerative medicine | | √ | |  | |  | |  | |  | | √ | |  | |
| MM | | Task 1.8 - Euro 300000 to study Parkinsons | |  | |  | |  | |  | | √ | | √ | |  | |
| MM | | Task 1.9 - Science elective | |  | |  | | √ | |  | | √ | | √ | |  | |
| MM | | Task 1.10 - Statistics and epidemiology (Y1) | |  | |  | |  | |  | | √ | | √ | |  | |
| MM | | Task 1.11 - Under the pressure | | √ | |  | |  | | √ | |  | |  | |  | |
| Second year | | | | | | | | | |  | |  | |  | |  | |
| SC | Task 2.1 - HNP, then and now | |  | |  | |  | |  | |  | | √ | | √ | |  |
| SC | Task 2.2 - Renal Insufficiency | |  | | √ | | √ | |  | |  | |  | |  | |  |
| SC | Task 2.3 - OrganICation and care around the patient with respiratory complaints | | √ | |  | |  | |  | |  | | √ | |  | |  |
| SC | Task 2.4 - Acid | |  | |  | |  | | √ | | √ | |  | |  | |  |
| SC | Task 2.5 - The smear, a close look | | √ | |  | |  | | √ | |  | |  | |  | |  |
| SC | Task 2.6 - Communication about menopause and sexuality | |  | | √ | |  | |  | |  | |  | | √ | |  |
| SC | Task 2.7 - Giving birth at home or in hospital | |  | |  | |  | |  | | √ | | √ | |  | |  |
| SC | Task 2.8 - Collaboration between general practitioner and specialist in the care of a sick child | |  | |  | | √ | |  | |  | |  | | √ | |  |
| SC | Task 2.9 - Alcohol and drugs, not so smart (if you were) | | √ | |  | |  | |  | | √ | |  | |  | |  |
| SC | Task 2.10 - Talking about the prostate | |  | | √ | |  | |  | | √ | |  | |  | |  |
| GH | Task 2.1 - Nervous system - Polio | | √ | | √ | | √ | |  | |  | | √ | |  | |  |
| GH | Task 2.2 - System diseases - Vitamins | | √ | |  | | √ | |  | | √ | | √ | |  | |  |
| GH | Task 2.3 - Short of breath | | √ | |  | | √ | | √ | | √ | |  | |  | |  |
| GH | Task 2.4 - Abdomen Hepatitis | |  | |  | | √ | |  | | √ | | √ | |  | |  |
| GH | Task 2.5a - Consultation skills | | √ | | √ | |  | |  | |  | |  | |  | |  |
| GH | Task 2.5b - Global Health Symposium | |  | |  | |  | |  | |  | | √ | | √ | |  |
| GH | Task 2.6a - FamiliarICation with Health Services | |  | |  | | √ | |  | |  | |  | | √ | |  |
| GH | Task 2.6b - International perspective on medical abortion | | √ | |  | |  | |  | | √ | |  | |  | |  |
| GH | Task 2.7 - Reproductive health - PICO-CAT | | √ | | √ | | √ | |  | |  | | √ | |  | |  |
| GH | Task 2.8a - Consultation skills | | √ | | √ | |  | |  | |  | |  | |  | |  |
| GH | Task 2.8b - Integrated management child illnesses | |  | |  | | √ | | √ | | √ | |  | |  | |  |
| GH | Task 2.9 - ADHD Child mental development | |  | | √ | |  | |  | |  | | √ | |  | |  |
| GH | Task 2.10 - Prostate cancer | |  | |  | |  | |  | | √ | | √ | |  | |  |
| IC | Task 2.1 - Nerves and senses | | √ | |  | |  | |  | | √ | |  | |  | |  |
| IC | Task 2.2 - Renal function and renal failure | |  | |  | |  | |  | | √ | | √ | |  | |  |
| IC | Task 2.3 - Diagnosing lung failure | | √ | |  | | √ | |  | |  | |  | |  | |  |
| IC | Task 2.4 - Diagnosis of abdominal complaints | | √ | | √ | |  | |  | |  | |  | |  | |  |
| IC | Task 2.5 - MDO Breast Carcinoma | |  | |  | |  | | √ | |  | |  | | √ | |  |
| IC | Task 2.6 - Endocrine disorders and gonadal function and dysfunction | | √ | | √ | |  | |  | |  | |  | |  | |  |
| IC | Task 2.7 - Evidence Based Medicine for the Clinic of O and G | |  | |  | |  | |  | | √ | | √ | |  | |  |
| IC | Task 2.8 - Diagnosis and treatment of pathological jaundice | | √ | | √ | |  | |  | |  | |  | |  | |  |
| IC | Task 2.9 - Etiological diagnosis of a child with developmental delay | |  | |  | | √ | | √ | |  | |  | |  | |  |
| IC | Task 2.10 - Talking about the prostate | |  | | √ | |  | |  | | √ | |  | |  | |  |
| MM | Task 2.1 - Biomarkers in health and disease | |  | |  | |  | |  | | √ | | √ | |  | |  |
| MM | Task 2.2 - Cancer immunotherapy | |  | | √ | |  | |  | |  | | √ | |  | |  |
| MM | Task 2.3 - Glaucoma dissected | | √ | |  | |  | |  | |  | | √ | |  | |  |
| MM | Task 2.4 - Oncologic imaging and treatment | | √ | |  | | √ | |  | |  | |  | |  | |  |
| MM | Task 2.5 - Shortness of breath | |  | | √ | |  | |  | | √ | |  | |  | |  |
| MM | Task 2.6 - Endocrine disorders and gonadal function and dysfunction | | √ | | √ | |  | |  | |  | |  | |  | |  |
| MM | Task 2.7 - First steps in child rehabilitation | | √ | |  | | √ | |  | |  | |  | |  | |  |
| MM | Task 2.8 - Healthy reproduction | |  | |  | |  | |  | | √ | |  | | √ | |  |
| MM | Task 2.9 - Prevention and treatment of sepsis in children | |  | | √ | |  | |  | | √ | |  | |  | |  |
| MM | Task 2.10 - Prostate cancer screening and treatment | |  | |  | |  | | √ | | √ | |  | |  | |  |

SC = Sustainable Care, IC = Intramural Care, GH = Global Health, MM = Molecular Medicine. MED = Medical Expert, COM = Communication, COL = Collaboration, LEA = Leadership, HA = Health advocate, SCH = Scholar, PRO = Professionalism.
